# Molecular Mechanisms of Animal Toxins, Venoms and Antivenoms

**DOI:** 10.3390/ijms242216389

**Published:** 2023-11-16

**Authors:** R. Manjunatha Kini, Yuri N. Utkin

**Affiliations:** 1 Department of Biological Sciences, National University of Singapore, Singapore 117558, Singapore; dbskinim@nus.edu.sg; 2 Laboratory of Molecular Toxinology, Shemyakin-Ovchinnikov Institute of Bioorganic Chemistry, 117997 Moscow, Russia

In many animals belonging to different taxa, venoms evolved as a means of defense and/or a means of attack/hunting. Venoms contain compounds of various chemical natures, usually called toxins. Over the course of evolution, toxins have acquired the ability to affect different systems in the organisms of prey, victims and predators. Usually, a single venom contains a large array of toxins directed against multiple biological targets. Depending on the species of the venomous animal, this array varies greatly. Toxins affect practically all vitally important systems in living organisms. Thus, in the nerve system, neurotoxins influence different stages of nerve impulse transmission [1,2], and in the cardiovascular system, toxins affect the heart, blood vessels and blood coagulation. The effects of some animal toxins result in muscle degradation [3]. The immune system is also influenced by these toxins; for example, complement is depleted by the cobra venom factor [4]. The manuscripts submitted to this Special Issue uncover the molecular mechanisms of animal toxin effects on the cardiovascular [5] and immune [6] systems.

The development of methods for the identification and analysis of the chemical structure of organic compounds [7] leads to the discovery of new toxins, for which it is necessary to establish the mechanisms of action. The work of Van Baelen et al. [8] published in this Special Issue is an example of the discovery of an animal toxin with new biological activity. Moreover, new species of venomous animals are being discovered, for which it is also necessary to establish the venom profiles and molecular mechanisms of venom action. Thus, mammalian representatives, including the platypus [9] and slow lorises [10], have recently been added to the animals traditionally considered venomous, such as snakes, scorpions and spiders.

Because animal toxins have evolved to interact with specific biological targets with high efficiency and selectivity, they are widely used as research tools. First, α-bungarotoxin from the venom of the krait *Bungarus multicinctus* should be mentioned [11]. The discovery of this toxin greatly contributed to the establishment of the molecular mechanisms of nerve impulse transduction [12]. Due to their high efficiency and selectivity of action, animal toxins are considered a promising basis for the creation of new drugs (e.g., [13,14]). However, despite numerous studies, the number of drugs used that are based on these toxins is currently quite limited [15].

Although not all animal venoms and toxins are dangerous to humans, the problem of treating bites from venomous animals remains a significant problem in a number of world regions [16]. Understanding the action mechanisms of animal venoms is very important for the effective treatment of intoxication by venomous animals. Currently, the most effective way to treat bites from venomous animals is the use of antisera, which is obtained by immunizing large mammals (mainly horses) with small doses of venom [17,18]. Although very effective, this method has several disadvantages and requires the development of new treatments based on other molecular mechanisms [19,20]. Without knowledge of the structures of toxins and their biological effects, this development is hardly possible.

All of the above factors dictate the need to study the structure and mechanisms of action of both animal toxins and venoms, as a source of new toxins.
